# The *Mycoplasma pneumoniae *MPN229 gene encodes a protein that selectively binds single-stranded DNA and stimulates Recombinase A-mediated DNA strand exchange

**DOI:** 10.1186/1471-2180-8-167

**Published:** 2008-10-02

**Authors:** Marcel Sluijter, Theo Hoogenboezem, Nico G Hartwig, Cornelis Vink

**Affiliations:** 1 Erasmus MC, Laboratory of Pediatrics, Pediatric Infectious Diseases, P.O. Box 2040, 3000 CA Rotterdam, the Netherlands

## Abstract

**Background:**

*Mycoplasma pneumoniae *has previously been characterized as a micro-organism that is genetically highly stable. In spite of this genetic stability, homologous DNA recombination has been hypothesized to lie at the basis of antigenic variation of the major surface protein, P1, of *M. pneumoniae*. In order to identify the proteins that may be involved in homologous DNA recombination in *M. pneumoniae*, we set out to characterize the MPN229 open reading frame (ORF), which bears sequence similarity to the gene encoding the single-stranded DNA-binding (SSB) protein of other micro-organisms.

**Results:**

The MPN229 ORF has the capacity to encode a 166-amino acid protein with a calculated molecular mass of 18.4 kDa. The amino acid sequence of this protein (*Mpn *SSB) is most closely related to that of the protein predicted to be encoded by the MG091 gene from *Mycoplasma genitalium *(61% identity). The MPN229 ORF was cloned, and different versions of *Mpn *SSB were expressed in *E. coli *and purified to > 95% homogeneity. The purified protein was found to exist primarily as a homo-tetramer in solution, and to strongly and selectively bind single-stranded DNA (ssDNA) in a divalent cation- and DNA substrate sequence-independent manner. *Mpn *SSB was found to bind with a higher affinity to ssDNA substrates larger than 20 nucleotides than to smaller substrates. In addition, the protein strongly stimulated *E. coli *Recombinase A (RecA)-promoted DNA strand exchange, which indicated that *Mpn *SSB may play an important role in DNA recombination processes in *M. pneumoniae*.

**Conclusion:**

The *M. pneumoniae *MPN229 gene encodes a protein, *Mpn *SSB, which selectively and efficiently binds ssDNA, and stimulates *E. coli *RecA-promoted homologous DNA recombination. Consequently, the *Mpn *SSB protein may play a crucial role in DNA recombinatorial pathways in *M. pneumoniae*. The results from this study will pave the way for unraveling these pathways and assess their role in antigenic variation of *M. pneumoniae*.

## Background

*Mycoplasma pneumoniae *is a human pathogen that causes a range of respiratory infections, such as tracheobronchitis, pharyngitis, and atypical pneumonia. *M. pneumoniae *causes up to 40% of community-acquired pneumonias and as many as 18% of cases requiring hospitalization in children (for a review, see [1]). It has previously been reported that *M. pneumoniae*-induced pneumonia may be observed most frequently among children 5–15 years of age. However, later studies have indicated that *M. pneumoniae *may also occur endemically and sometimes epidemically in older persons as well as in children less than 5 years of age.

Mycoplasmas are among the smallest self-replicating organisms, in both cellular dimensions and genome size, that are capable of free existence [2]. The genome of *M. pneumoniae *(strain M129) was found to have a length of 816,394 base pairs (bp), containing 688 open reading frames (ORFs) [3,4]. As would be expected on the basis of the relatively small size of its genome, being at least 5 times smaller than the *Escherichia coli *genome, *M. pneumoniae *possesses limited metabolic and biosynthetic capacities in comparison to 'classical' bacteria [5]. These limitations necessitate a parasitic lifestyle, and a close association of the bacterium with the respiratory epithelium of its human host.

A crucial step in the initiation of infection by *M. pneumoniae *is its attachment to the respiratory epithelium (cytadherence) of the host. This process is essential to pathogenesis since mutants that are unable to adhere are also avirulent [6]. Cytadherence is mediated by a specialized and complex attachment organelle. This organelle is localized at the tip of the bacterium and consists of a network of adhesins and accessory proteins. The major adhesion protein (cytadhesin) that is concentrated in the attachment organelle is the surface-exposed, 170-kDa P1 protein. This protein, which is encoded by the MPN141 gene, was demonstrated to be essential for attachment [6].

Apart from its function in cytadherence, the P1 protein is known to elicit a strong humoral immune response during infection [7]. In relation to this immunodominance, the P1 protein was hypothesized to undergo antigenic variation [8]. At the basis of this hypothesis is the finding within the P1 gene of two sequence blocks of which multiple variants exist throughout the *M. pneumoniae *genome [9,10]. These sequences (repetitive elements) were designated RepMP4, which is located near the 5' end of the P1 gene, and RepMP2/3, which is located at the 3' end [11]. Within the *M. pneumoniae *M129 genome, a total of 8 variants of RepMP4 and 10 variants of RepMP2/3 were identified [3]. Because the different variants of a given RepMP element are closely related in sequence, but not identical, it is possible that recombination between the P1 gene and RepMP sequences elsewhere in the genome could generate significant sequence variation within the P1 gene, resulting in amino acid changes in the P1 protein at the bacterial surface.

In order to demonstrate that recombination between RepMP elements lies at the heart of antigenic variation of *M. pneumoniae*, we initiated a study aimed at the identification of proteins that may be involved in this process. On the basis of sequence comparison, at least four *M. pneumoniae *ORFs were proposed to play a role in DNA recombination: MPN229 (or G07_orf166), MPN490 (or P02_orf336), MPN535 (or G12_orf206) and MPN536 (or G12_orf307), which may encode homologs of the *E. coli *single-stranded DNA binding protein (SSB), RecA, RuvA and RuvB, respectively [3]. In *E. coli*, SSB is a protein that primarily binds single-stranded DNA (ssDNA) and plays a central role in most aspects of DNA metabolism, including DNA replication and recombination. *E. coli *RecA is responsible for promoting pairing between a single-stranded donor DNA molecule and a homologous, double-stranded recipient DNA molecule, while RuvA and RuvB interact to form RuvAB, which possesses DNA helicase activity and catalyzes branch formation during recombination of two DNA strands.

Of the *M. pneumoniae *ORFs that were predicted to encode proteins involved in DNA recombination, only MPN535 has been studied in detail [12]. This protein (*Mp*RuvA) was demonstrated to bind Holliday junctions and other branched DNA substrates in a similar fashion as *E. coli *RuvA [12]. In order to initiate the analysis of the complete set of putative DNA recombination enzymes from *M. pneumoniae*, we have examined the characteristics of the MPN229 protein product, which was designated *Mpn *single-stranded DNA-binding protein (*Mpn *SSB). We here report that *Mpn *SSB has a strong and selective ssDNA-binding activity, and promotes *E. coli *RecA-dependent DNA strand transfer.

## Methods

### Strains

*M. pneumoniae *strain MAC (ATCC^® ^no. 15492™) was cultured in Mycoplasma medium containing 1.4% Difco™ PPLO broth (Becton Dickinson), 0.15% Difco™ TC Yeastolate, UF (Becton Dickinson), 1.4% glucose, 20% horse serum, 1,000 U/ml Penicillin G, 500 U/ml Polymyxine B, 3 μg/ml Fungizone, 5 μg/ml Voriconazol and 0.02 mg/ml phenol red. The pH of the medium was adjusted to 7.8–8.0 using a solution of 2 N NaOH. Finally, the medium was filter-sterilized. Cells were harvested upon color change of the medium (from red/orange to yellow).

### Cloning of the MPN229 gene and generation of Mpn SSB expression constructs

DNA was purified from cultures of *M. pneumoniae *as follows. Cultures (3 ml) were harvested by centrifugation for 10 min in a microcentrifuge at full speed. The cell pellet was resuspended in 1 ml 0.9% NaCl. After centrifugation, the cell pellet was resuspended in 400 μl of TE buffer (10 mM Tris-HCl, 1 mM EDTA pH 8.0). Then, 70 μl of 10% sodium dodecyl sulfate (SDS) and 5 μl of Proteinase K (10 mg/ml) was added. After incubation for 10 min at 65°C, 100 μl of 5 M NaCl was added, followed by the addition of 100 μl of a solution of 10% N-cetyl-N,N,N-trimethylammonium bromide (CTAB) in 0.7 M NaCl. The solution was incubated for 10 min at 65°C. The DNA was subsequently extracted from the solution using 500 μl of chloroform/isoamyl alcohol (24:1, vol:vol). The DNA in the upper, aqueous phase was transferred to a fresh tube and precipitated by the addition of 360 μl of isopropanol. Following incubation at -20°C for 30 min, the DNA was pelleted by centrifugation for 10 min at full speed in a microcentrifuge. The DNA pellet was washed with 70% ethanol, dried, and resuspended in 10 μl of H_2_O. The MPN229 (or G07_orf166 [3]) ORF was amplified by PCR from the *M. pneumoniae *MAC genomic DNA. The PCR mixture (50 μl) contained 0.3 μM of oligonucleotide primer SSB_fw (5'-GGTCGT**CAT*ATG***AACCGCGTTTTTTTATTTG-3'; the sequence in bold indicates a unique *Nde*I restriction endonuclease recognition site, which overlaps with the translation initiation codon [in italics] of MPN229), 0.3 μM of primer SSB_rv (5'-GCAGCC**GGATCC***TTA*TTCATCATCACTCTCCTC-3'; the sequence in bold indicates a unique *Bam*HI restriction site, which is adjacent to the translation termination codon [anticodon in italics] of MPN229), 10 ng of *M. pneumoniae *genomic DNA, 0.2 mM of each dNTP, 1 unit of *Pfu *DNA polymerase (Fermentas), and 1 × *Pfu *buffer containing MgSO_4 _(20 mM Tris-HCl [pH 8.8], 10 mM (NH_4_)_2_SO_4_, 10 mM KCl, 0.1% Triton X-100, 0.1 mg/ml BSA, and 2 mM MgSO_4_). PCR was performed using the following conditions: 3 min at 95°C, followed by 30 cycles of 30 sec at 95°C, 30 sec at 50°C, and 2 min at 72°C. The resulting 522-base pairs (bp) PCR fragment was cloned into the pJET1/blunt cloning vector (Fermentas) using the GeneJET™ PCR cloning kit (Fermentas). From the generated plasmid, pJET1-*Mpn*SSB, the MPN229 ORF was excised by digestion with *Nde*I and *Bam*HI, and cloned into *Nde*I- and *Bam*HI-digested expression vectors pET-11c and pET-16b (Novagen), resulting in plasmid pET-11c-*Mpn*SSB and pET-16b-*Mpn*SSB, respectively. In plasmid pET-11c-*Mpn*SSB, the MPN229 ORF is cloned such as to express *Mpn *SSB in its natural, non-tagged form. In plasmid pET-16b-*Mpn*SSB, the MPN229 ORF is fused at its 5' end to a sequence encoding a polyhistidine tag (MG(H)_10_SSGHIEGRH). This polyhistidine sequence allows one-step affinity purification of the MPN229-encoded protein by Ni^2+^-affinity chromatography (see below). For the generation of a glutathione S-transferase (GST)-*Mpn *SSB fusion protein expression construct, the *Nde*I-*Bam*HI fragment containing MPN229 was first made blunt-ended by incubation with dNTPs and 'Klenow fragment' (Fermentas) and then cloned into *Sma*I-digested and Shrimp alkaline phosphatase (Promega)-treated vector pRP265 (NCCB Plasmids database, element No. PC-V3271; ). Plasmid pRP265 is a derivative of the expression plasmid pGEX-2T in which the polylinker 5'-GGATCCCCGGGAATTC-3' has been replaced by the sequence 5'-GGATCCCCATGGTACCCGGGTCGACTAGTATGCATAAGCTTGAATTC-3'. The resulting plasmid was termed pRP265-*Mpn*SSB. Vector pRP265 was used for the production of native GST. The integrity of all DNA constructs used in this study was verified by DNA sequencing.

### Expression and purification of Mpn SSB, Mpn H_10_-SSB and GST(-SSB)

Constructs pET-11c-*Mpn*SSB, pET-16b-*Mpn*SSB, pRP265-*Mpn*SSB and pRP265 were introduced into *E. coli *BL21(DE3) and the resulting strains were grown overnight at 37°C in LB medium containing 100 μg/ml ampicillin. The cultures were diluted 1:100 in 100–150 ml LB medium with ampicillin and grown at 37°C to an optical density at 600 nm of 0.6. Protein expression was then induced by the addition of isopropyl-β-D-thiogalactopyranoside (IPTG) to a final concentration of 0.3 mM. After 3 hr, the bacteria were harvested by centrifugation and stored at -20°C.

Non-tagged *Mpn *SSB was purifed as follows. Frozen, bacterial pellets corresponding to 100 ml of culture were resuspended in 5 ml of buffer A (20 mM Tris-HCl pH 7.5, 150 mM NaCl, 1 mM DTT). The suspension was sonicated on ice and clarified by centrifugation for 20 min at 12,000 × *g *(4°C). To the supernatant, 5 ml of saturated ammonium sulphate was added, followed by 15 min of incubation on ice. After centrifugation for 10 min at 10,000 × g (4°C), the protein pellet, which contained the *Mpn *SSB protein, was dissolved in 10 ml of buffer A. Subsequently, 1 ml of a 50% suspension of DNA-cellulose (Worthington biochemical corp., Lakewood NJ), equilibrated in buffer A, was added, and the suspension was left on a rotating mixer for 1 hour at 4°C. The DNA-cellulose was poured into a column, and protein was eluted stepwise by the addition of 0.5 ml of a buffer (20 mM Tris-HCl pH 7.5, 1 mM DTT) containing increasing concentrations of NaCl (from 200 to 1500 mM). The fractions containing *Mpn *SSB were pooled and dialyzed against 20 mM Tris-HCl pH 7.4, 150 mM NaCl, 0.1 mM EDTA, 1 mM DTT, 0.1% Tween 20, 50% glycerol. The protein was stored at -20°C.

The polyhistidine-tagged *Mpn *SSB protein (*Mpn *H_10_-SSB) was purified as follows. Bacterial pellets corresponding to 150 ml of culture were resuspended in 10 ml of buffer B (20 mM Tris-HCl pH 8.0, 0.3 M NaCl) plus 5 mM imidazole. The suspension was sonicated on ice and clarified by centrifugation for 20 min at 12,000 × *g *(4°C). To the supernatant, 1 ml was added of a 50% slurry of Ni^2+^-nitroloacetic acid (Ni-NTA)-agarose (Qiagen), equilibrated previously in buffer B containing 5 mM imidazole. The suspension was stirred for 1 hr at 4°C and subsequently poured into a column. Non-specifically bound proteins were washed from the Ni-NTA agarose by two subsequent washes (of 4 ml each) with buffer B containing 5 mM, 10 mM and 20 mM imidazole, respectively. The bound *Mpn *SSB protein was eluted from the column with 2.5 ml of buffer B containing 250 mM imidazole. Fractions of 0.5 ml were collected, analyzed by SDS-polyacrylamide gel electrophoresis (SDS-PAGE), pooled, and dialyzed against buffer B plus 1 mM DTT and 50% glycerol. Aliquots of purified protein were stored at -20°C.

Both GST and GST-tagged *Mpn *SSB protein (GST-SSB) were purified as follows. Bacterial pellets corresponding to 100 ml of culture were resuspended on ice in 5 ml of PBS (140 mM NaCl, 2.7 mM KCl, 10 mM Na_2_HPO_4_, 1.8 mM KH_2_PO_4_, pH 7.3) and sonicated. Then, Triton X-100 was added to a final concentration of 1%, and the suspension was left on a rotating mixer for 30 min at 4°C. Subsequently, the suspension was clarified by centrifugation for 20 min at 10,000 × *g *(4°C). To the supernatant, 0.8 ml was added of a 50% slurry of Glutathione-Agarose (Sigma, Saint Louis, MO, USA), equilibrated previously in PBS. The suspension was incubated on a rotating mixer for 30 min at 4°C and subsequently poured into a column. The column was washed consecutively with 3 ml of PBS containing 1% Triton X-100 and with 5 ml of PBS. Specifically bound protein was eluted from the column by addition of 3 ml of 50 mM Tris-HCl pH 8.0 containing 10 mM reduced glutathione (Sigma). Fractions of 0.5 ml were collected, analyzed by SDS-PAGE, pooled, and dialyzed against 20 mM Tris-HCl pH 7.4, 150 mM NaCl, 0.1 mM EDTA, 1 mM DTT, 0.1% Tween 20, 50% glycerol. Aliquots of purified protein were stored at -20°C. Protein concentrations were determined using the BC Assay protein quantitation kit (Interchim) as well as by SDS-PAGE analysis in conjunction with bovine serum albumin standards. All purified proteins were obtained at concentrations of at least 1 mg/ml, having an estimated homogeneity of 95% or greater. The concentrations of all proteins used in this study refer to monomeric protein concentrations throughout the manuscript.

### SDS-PAGE

Proteins were separated on SDS-polyacrylamide gels, essentially as described by Laemmli [13]. Following electrophoresis, gels were stained with Coomassie brilliant blue (CBB), destained in 40% methanol/10% acetic acid, and photographed using a GelDoc XR system (Bio-Rad). Digital images were processed using Quantity One^® ^1-D Analysis Software (Bio-Rad).

### Gel filtration chromatography

Gel filtration chromatography was carried out by applying a sample of 500 μl of *Mpn *SSB (at 0.3 mg/ml in 50 mM Tris-HCl (pH 7.5), 135 mM NaCl) to a Sephadex G-150 column with a length of 1 m and an inner diameter of 1.0 cm. The column was eluted with 50 mM Tris-HCl (pH 7.5), 135 mM NaCl at an elution rate of 4 ml/h. The column was calibrated with bovine serum albumin (BSA, 66.4 kDa), ovalbumin (42.9 kDa), and cytochrome C (12.3 kDa). Void volume was determined with blue dextran (2,000 kDa). Fractions of 1.0 ml were collected and analyzed by measuring the optical density at 280 nm (OD_280_). Since a relatively low concentration of *Mpn *SSB was loaded on the column, the eluted fractions were precipitated with trichloroacetic acid, and separated on 14% SDS-PAGE gels. Gels were stained with CBB and recorded using the GelDoc XR system (Bio-Rad). In fractions that contained *Mpn *SSB, the amount of protein was determined semi-quantitatively using BioNumerics Version 3.0 software (Applied Maths).

### DNA substrates

The sequences of the oligonucleotide substrates that were used in this study are listed in Table 1. Oligonucleotides were purchased from Eurogentec and were used either unlabeled or 5'-end labeled with ^32^P. Bacteriophage M13mp18 circular, single-stranded DNA was purified from cultures of infected *E. coli *XL-Blue cells (Stratagene), essentially as described previously [14]. Bacteriophage φX174 RF1 DNA was purchased from Fermentas. The DNA was linearized by digestion with *Pst*I (Invitrogen), followed by inactivation of the restriction enzyme by incubation for 20 min at 80°C. Viron DNA from φX174 was purchased from New England Biolabs. The concentration of bacteriophage-derived DNAs was determined using the NanoDrop 1000 (Thermo Scientific). The DNA concentrations are indicated for DNA molecules and not for nucleotides for all substrates throughout the manuscript.

**Table 1:** Sequences of the oligonucleotide substrates used in this study

Name	Sequence
Oligo 1 (48-mer)	5'-GGAACAGCTACAGCTGATCATCACCATCACCATCACTAGGATCCGCAT-3'
Oligo 2 (49-mer)	5'-CACGTGCTAGCCATCACCATCACCATCACGTGAAGACAACAGTCTATCC-3'
Oligo 3 (50-mer)	5'-CAGGTGCACCAGAACAACCACATCACCATCACCATCACTAGGATCCGCAT-3'
	
(A)_50 _(50-mer)	5'-AAAAAAAAAAAAAAAAAAAAAAAAAAAAAAAAAAAAAAAAAAAAAAAAAA-3'
(T)_50 _(50-mer)	5'-TTTTTTTTTTTTTTTTTTTTTTTTTTTTTTTTTTTTTTTTTTTTTTTTTT-3'
(C)_50 _(50-mer)	5'-CCCCCCCCCCCCCCCCCCCCCCCCCCCCCCCCCCCCCCCCCCCCCCCCCC-3'
(G)_50 _(50-mer)	5'-GGGGGGGGGGGGGGGGGGGGGGGGGGGGGGGGGGGGGGGGGGGGGGGGGG-3'
	
15 nt (15-mer)	5'-CCCTGTTAGCACTGT-3'
20 nt (20-mer)	5'-CCCTGTTAGCACTGTTGTAT-3'
30 nt (30-mer)	5'-CCCTGTTAGCACTGTTGTATCCAGCATCCT-3'
40 nt (40-mer)	5'-CCCTGTTAGCACTGTTGTATCCAGCATCCTGTCTTGCAGA-3'
50 nt (50-mer)	5'-CCCTGTTAGCACTGTTGTATCCAGCATCCTGTCTTGCAGAGCATTCCGAC-3'

### DNA-binding assays

DNA-binding experiments were carried out with either oligonucleotide substrates (Table 1), bacteriophage M13mp18 DNA, or bacteriophage φX174 DNA (both single- and double-stranded). Reactions were performed in volumes of 20 μl with various concentrations of purified protein in 25 mM Tris-OAc pH 7.5, 10 mM Mg(OAc)_2_, 1 mM DTT, 5% glycerol, and either a single-stranded (ss) unlabeled oligonucleotide (5 μM), 5'-^32^P-labeled oligonucleotide (1 μM), double-stranded (ds) supercoiled bacteriophage DNA (M13mp18 or φX174 DNA) (1 nM), or ss bacteriophage DNA (M13mp18 or φX174 DNA) (2 nM). After incubation for 15 min at 37°C, 2 μl was added of a solution containing 40% glycerol and 0.25% bromophenol blue. Subsequently, the samples were electrophoresed in 0.5 × TBE buffer, either through 1.0% agarose gels (when using unlabeled oligonucleotides), 0.6% agarose gels (when using bacteriophage DNA) or 5% polyacrylamide gels (when using ^32^P-labeled oligonucleotides). Agarose gels were stained with ethidium bromide and photographed. The polyacrylamide gels were subjected to autoradiography.

### Three-strand transfer assay

*E. coli *RecA-promoted DNA strand transfer reactions were carried out using φX174 DNA, in a similar fashion as described previously [15,16]. Reactions (30 μl) contained 70 mM Tris-HCl pH 7.6, 10 mM MgCl_2_, 5 mM DTT, 5 mM ATP, 0.08 U/μl Pyruvate Kinase Preparation (Type VII, from rabbit muscle [Sigma]), 3.2 mM phospho(enol)pyruvic acid (Sigma), 1 nM ss circular φX174 DNA, 2 nM *Pst*I-digested, ds φX174 DNA, 0.2 μg/μl *E. coli *Rec A (New England Biolabs), and 3 ng/μl of either *E. coli *SSB (Epicentre Biotechnologies), or 6.5 ng/μl of either *Mpn *SSB, GST-SSB or GST. Samples of 10 μl were taken at 0, 30 and 60 min of incubation at 37°C, and reactions were terminated by the addition of 1 μl of a solution of 5% SDS and 50 mM EDTA pH 8.0, followed by incubation for 10 min at 55°C. After the addition of loading dye, the samples were separated on 0.6% agarose gels in 0.5 × TBE. The gels were stained with ethidium bromide and photographed.

## Results

### Coding capacity of the MPN229 ORF

**Figure 1:**
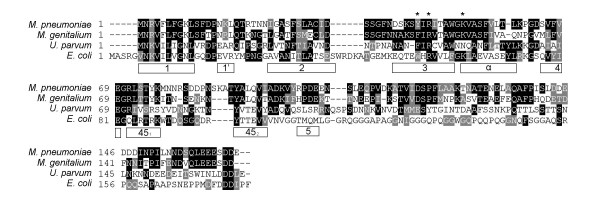
**Multiple alignment of the amino acid sequences predicted to be encoded by the *M. pneumoniae *MPN229 **[3]**, *M. genitalium *MG091**[17]**, *U. parvum ssb ***[39]**, and *E. coli ssb ***[40]** genes**. The amino acid motifs that are characteristic for the OB DNA-binding fold are indicated by the open boxes below the aligned sequences [18]. The asterisk above Y44 of the *M. pneumoniae *sequence indicates the position of a Trp residue (W55) in *E. coli *SSB that was shown to play a role in DNA-binding. The other two asterisks indicate conserved arginine (R) and lysine (K) residues (R46 and K52, respectively, in the *M. pneumoniae *sequence); for *E. coli *SSB these residues were shown to be oriented toward the DNA binding cleft [32]. The multiple aligment was performed using Clustal W . The program BOXSHADE 3.21  was used to generate white letters on black boxes (for residues that are identical in at least two out of four sequences) and white letters on grey boxes (for residues that are similar in at least two out of four sequences).

The *M. pneumoniae *MPN229 ORF was recognized as an ORF putatively encoding a homolog of single-stranded DNA binding proteins after determination of the complete genome sequence of *M. pneumoniae *strain M129 [3]. MPN229 has the capacity to encode a 166-amino acid protein (termed *Mpn *SSB) with a theoretical molecular mass of 18.4 kDa. The predicted amino acid sequence of *Mpn *SSB displays only limited similarity with the protein encoded by the SSB gene from *E. coli *(17% identity). A significantly higher similarity was seen with the protein that is putatively encoded by the MG091 gene [17] from *Mycoplasma genitalium *(61% identity). The residues that are shared among the predicted amino acid sequences of the (putative) SSB proteins from *M. pneumoniae*, *M. genitalium*, *Ureaplasma parvum *(all belonging to the family of Mycoplasmataceae) as well as from *E. coli*, are depicted in a multiple sequence alignment in Fig. 1. Most bacterial SSB proteins that have been studied to date possess a so-called oligonucleotide/oligosaccharide-binding (OB) fold and function as homotetramers [18,19]. By contrast, the SSB proteins from *Thermus aquaticus*, *Thermus thermophilus *[20] and *Deinococcus radiodurans *[21], which have a significantly higher molecular weight than the *E. coli *(-like) SSB protein(s), were reported to contain two OB folds per monomer and function as homodimers. As shown in Fig. 1, *Mpn *SSB, which is 12 amino acids smaller than the 178-amino acid *E. coli *SSB, may contain a single OB fold. The amino acid motifs that are typical of the OB fold are indicated by the open boxes below the aligned sequences in Fig. 1.

### Salient features of the Mpn SSB sequence

The C-terminus of the *Mpn *SSB sequence contains a high proportion of acidic amino acid residues: 13 of the 24 C-terminal amino acids are aspartic acid (D) or glutamic acid (E) residues. Albeit less extensive, acidic residues are also concentrated near the C-terminus of other SSB proteins. In *E. coli*, these residues have been demonstrated to be involved in the interaction of SSB with other proteins, such as exonuclease I, uracil DNA glycosylase, and DNA polymerase III [22-30]. Several residues of the *E. coli *SSB protein have been demonstrated to play a role in DNA-binding. These include three tryptophan (W) residues at position 41 (adjacent to OB box '2' in Fig. 1), 55 (within box '3') and 89 (adjacent to box '45_1_') [31]. However, none of these residues appear to be conserved with the SSB proteins from the Mycoplasmataceae. Only W55 seems to have a clear counterpart in the mycoplasma proteins, i.e. a tyrosine (Y) residue in *M. pneumoniae *(Y44) and a phenylalanine (F) in both *M. genitalium *(F44) and *U. parvum *(F43). Other residues that were found to be implicated in *E. coli *SSB DNA-binding, however, do have a counterpart in the mycoplasma SSBs. The arginine (R) and lysine (K) residues at positions 57 and 63, respectively, of *E. coli *SSB, which were shown to be oriented toward the DNA binding cleft [32], are conserved in the *M. pneumoniae *and *M. genitalium *SSB proteins (R46 and K52, respectively, in both proteins). These residues are located in the predicted OB fold motifs '3' and 'α' (Fig. 1). Interestingly, the histidine (H) residue that was described to be important for tetramerization of *E. coli *SSB (H56, central to OB fold box '3') [33] is not conserved among the proteins listed in Fig. 1; an isoleucine (I) is present at a position congruent to that of H56 in the mycoplasma proteins (residue I45 in *M. pneumoniae *SSB). The lack of conservation of this apparently crucial amino acid residue, however, can be reconciled with the finding that substitution of residue H56 of *E. coli *SSB for either an F or I residue, did not change the properties of the protein measurably [33].

### Expression and purification of the MPN229-encoded protein

**Figure 2:**
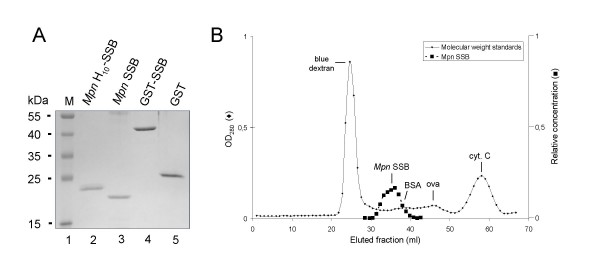
**Purification of recombinant *Mpn *SSB proteins**. (A) Samples of purified recombinant *Mpn *SSB proteins, i.e. *Mpn *H_10_-SSB (lane 2), *Mpn *SSB (lane 3), GST-SSB (lane 4), and purified GST (lane 5), were analyzed by SDS-PAGE (12%) and Coomassie brilliant blue (CBB)-staining. The sizes of protein markers (lane 1; PageRuler™ Prestained Protein Ladder [Fermentas]) are shown on the left-hand side of the gel. (B) Gel filtration analysis of *Mpn *SSB. Gel filtration chromatography was performed by applying *Mpn *SSB to a Sephadex G-150 column. The column was calibrated with blue dextran (2,000 kDa), bovine serum albumin (BSA, 66.4 kDa), ovalbumin (ova, 42.9 kDa), and cytochrome C (cyt. C, 12.3 kDa). Fractions of 1.0 ml were collected and monitored by measuring the optical density at 280 nm (OD_280_, Y-axis at the left-hand side of the graph). The fractions eluted from the subsequent run containing *Mpn *SSB were precipitated with trichloroacetic acid, and separated on 14% SDS-PAGE gels. Gels were stained with CBB and recorded using the GelDoc XR system (Bio-Rad). In fractions that contained *Mpn *SSB, the amount of protein was determined semi-quantitatively using BioNumerics Version 3.0 software (Applied Maths). The relative concentration of *Mpn *SSB (Y-axis on the right-hand side, in arbitrary units) in these fractions is plotted. In all other fractions, *Mpn *SSB was not detected by SDS-PAGE analysis and Coomassie brilliant blue-staining.

The MPN229 gene was cloned into different expression vectors in order to express *Mpn *SSB either in its native form (*Mpn *SSB), as an N-terminal polyhistidine-tagged polypeptide (*Mpn *H_10_-SSB) or as a glutathione S-transferase (GST)-tagged fusion protein (GST-SSB). Each of these proteins was expressed to high levels in *E. coli *and was purified to near homogeneity (Fig. 2A). The estimated molecular weights of the purified proteins corresponded to their theoretical molecular weights of 18.4 kDa, 20.9 kD and 45.0 kDa for *Mpn *SSB, *Mpn *H_10_-SSB, and GST-SSB, respectively. As a control, we also purified non-fused GST (Fig. 2A, lane 5). While most experiments were performed using *Mpn *H_10_-SSB, the activities of the three different SSB variants were found to be indistinguishable.

### The oligomeric form of Mpn SSB in solution

In order to determine the oligomeric state of *Mpn *SSB in solution, the untagged, purified protein was subjected to size-exclusion chromatography. As shown in Fig. 2B, *Mpn *SSB eluted from the column as a single, major protein species with an estimated molecular weight of ~75 kDa. This molecular weight corresponds to the theoretical molecular weight of the tetrameric form of *Mpn *SSB (73.6 kDa). These data therefore suggests that *Mpn *SSB exists predominantly as a homo-tetramer in solution. A similar oligomeric composition has previously also been found for SSB proteins from other bacterial species, such as *E. coli *[32].

### Oligonucleotide binding by Mpn SSB

**Figure 3:**
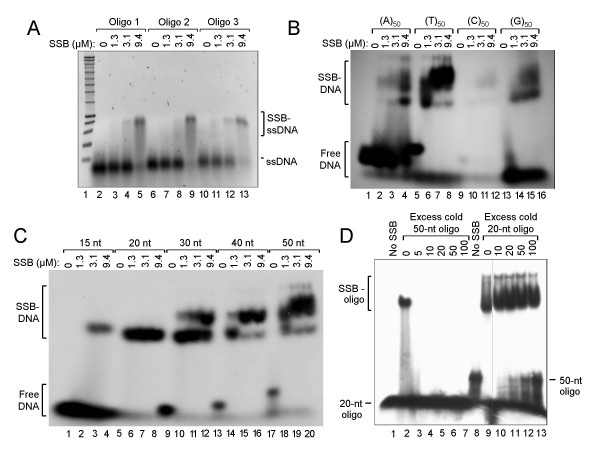
**The *Mpn *SSB protein binds oligonucleotide substrates in a DNA sequence-independent fashion**. (A) Binding of *Mpn *H_10_-SSB to three different oligonucleotide substrates. Reactions were performed in volumes of 20 μl and contained either 0 μM (lanes 2, 6 and 10), 1.3 μM (lanes 3, 7 and 11), 3.1 μM (lanes 4, 8 and 12) or 9.4 μM (lanes 5, 9 and 13) of *Mpn *H_10_-SSB, and 5 μM of either of three different single-stranded oligonucleotides (Oligo 1, 2 or 3; Table 1). After incubation for 15 min at 37°C, the samples were electrophoresed on a 1.0% agarose gel in 0.5 × TBE buffer. A black/white inverted image of a typical ethidium bromide-stained gel is shown. (B) Binding of *Mpn *H_10_-SSB (at 0, 1.3, 3.1 or 9.4 μM in lanes 1–4, 5–8, 9–12 and 13–16, respectively) to 5'-^32^P-labeled homooligomeric DNA substrates (at 1 μM; Table 1). The samples were separated on 5% polyacrylamide gels in 0.5 × TBE buffer. An autoradiograph is shown. (C) Binding of *Mpn *H_10_-SSB (at 0, 1.3, 3.1 or 9.4 μM) to a series of 15- to 50-mer 5'-^32^P-labeled oligonucleotides (at 1 μM), each containing the same 15-nucleotide core sequence (Table 1). The samples were processed as described above in (B). (D) A DNA-binding competition experiment in which *Mpn *H_10_-SSB (at 3.1 μM) was incubated with a constant amount (1 μM) of either the 5'-^32^P-labeled '20 nt' oligonucleotide (lanes 1–7) or '50-nt' oligonucleotide (lanes 8–13) (Table 1), and increasing amounts of the other, unlabeled ('cold') oligonucleotide. A molar excess of 5 to 100 times unlabeled oligonucleotide over labeled oligonucleotide was tested, as indicated above the lanes. From the samples loaded in lanes 1 and 8, *Mpn *H_10_-SSB was omitted; the gel was processed similarly as in (B).

To investigate the potential of *Mpn *H_10_-SSB to bind DNA, the protein was incubated at various concentrations with three different single-stranded oligonucleotide (ssDNA) substrates. The resulting complexes were analyzed by non-denaturing agarose gel electrophoresis, followed by staining with ethidium bromide. As shown in Fig. 3A, an increasing amount of product with a lower mobility than that of the free oligonucleotides was generated with increasing *Mpn *H_10_-SSB concentrations. At the highest protein concentration tested (9.4 μM; lanes 5, 9 and 13), virtually all of the ssDNA formed part of the lower mobility complex, which was assumed to represent *Mpn *H_10_-SSB-ssDNA complexes. DNA-binding by *Mpn *H_10_-SSB was found to be rapid, as the DNA-protein complexes shown in Fig. 3A were already produced in less than 1 min of incubation at 37°C (data not shown). Similar results were obtained with the other recombinant SSB proteins (data not shown).

Because protein-DNA complexes were generated with each of the three different ssDNA substrates tested, we hypothesized that *Mpn *SSB may bind ssDNA in a sequence-independent manner. To further investigate this, we tested the ability of *Mpn *H_10_-SSB to bind ^32^P-labeled homooligomers (50-mers) of dA, dT, dC and dG (Table 1). Although the (T)_50 _substrate seemed to be bound most efficiently, each of the homooligomers was bound by the protein (Fig. 3B). The (C)_50 _substrate, despite being labeled inefficiently, most likely due to the formation of inaccessible secondary structures, was also bound by *Mpn *H_10_-SSB. These data show that the protein indeed binds ssDNA irrespective of the DNA substrate sequence. It is interesting to note that binding of *Mpn *H_10_-SSB to the 50-nucleotide homooligomer substrates resulted in the formation of two different DNA-protein complexes (Fig. 3B). As the formation of the larger of the two complexes was favored at higher protein concentrations, it is likely that this larger species represents DNA-protein complexes in which a higher number of SSB DNA-binding units (eg., dimers or tetramers) are present than in the smaller species. Thus, a 50-mer DNA substrate may be bound by a single binding unit at relatively low protein concentrations, whereas two or more binding units may occupy the substrate at higher protein concentrations. To study this further, we tested binding of *Mpn *H_10_-SSB to a series of oligonucleotides with lengths ranging from 15 to 50 nucleotides, and each containing the same 15-nucleotide 'core sequence', which was randomly chosen (Table 1). As shown in Fig. 3C, only the 15-nucleotide substrate was bound inefficiently by the protein, whereas the other substrates were bound efficiently, each being completely complexed at higher protein concentrations. Interestingly, a second, larger DNA-protein complex was seen exclusively with substrates with a length equal to or larger than 30 nucleotides. The efficiency of formation of these larger complexes was even higher with the 40- or 50-mer substrates (lanes 14–20). Thus, whereas a 20-mer substrate may accommodate binding of a single SSB DNA-binding unit, longer substrates may allow binding of two or more units, generating protein-DNA complexes with a lower electrophoretic mobility than do single unit-bound complexes.

To investigate whether *Mpn *SSB binds with similar affinities to 20- and 50-mer substrates, a DNA-binding competition experiment was performed in which *Mpn *H_10_-SSB was incubated with a constant amount of radiolabeled oligonucleotide and increasing amounts of the other, unlabeled oligonucleotide (Fig. 3D). Complexes with 20-mer substrates were no longer observed when a 5-fold molar excess of the 50-mer substrate was present in the DNA-binding reaction (compare lanes 2 and 3). By contrast, the formation of complexes with the 50-mer substrate was only partially inhibited in the presence of a 20-100-fold excess of unlabeled 20-mer oligonucleotide (Fig. 3D, lanes 11–13). These results indicate that *Mpn *H_10_-SSB binds with a higher affinity to the 50-mer substrate than to the 20-mer substrate.

### dsDNA- versus ssDNA-binding of SSB and divalent cation-dependence of DNA-binding

**Figure 4:**
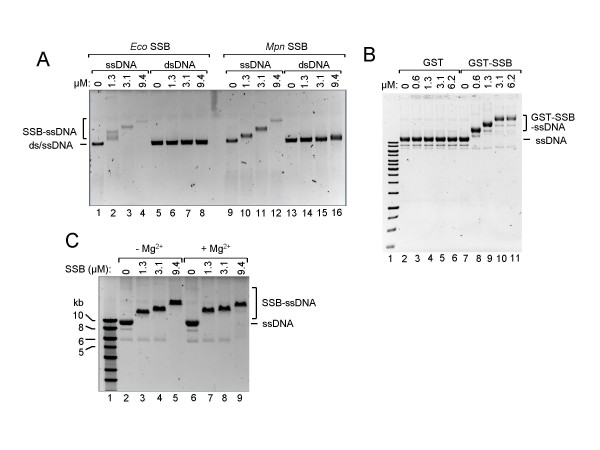
***Mpn *SSB binding to long DNA substrates and Mg^2+^-dependence of DNA-binding**. (A) Binding of *E. coli *SSB (*Eco *SSB, lanes 1–8) and *Mpn *SSB (lanes 9–16) to either 2 nM of ss φX174 DNA (ssDNA, lanes 1–4 and lanes 9–12) or 1 nM of ds φX174 DNA (dsDNA, lanes 5–8 and lanes 13–16). Reactions were performed in volumes of 20 μl and contained various concentrations of protein, as indicated above the lanes. The samples were separated on a 0.6% agarose gel in 0.5 × TBE buffer. A black/white inverted image of a typical ethidium bromide-stained gel is shown. (B) Binding of GST-SSB to circular, single-stranded M13mp18 DNA (ssDNA). Binding reactions were carried out with various concentrations of either GST-SSB (lanes 8–11) or GST (lanes 3–6), as indicated above the lanes, and 2 nM of DNA. The samples were separated on 0.6% agarose gels in 0.5 × TBE buffer and processed as described above. (C) DNA-binding by *Mpn *SSB is Mg^2+^-independent. Reactions with *Mpn *H_10_-SSB were executed similarly as described in (B), except for the omission of Mg(OAc)_2 _in the reaction mixtures of the samples loaded in lanes 2–5. Complexes of *Mpn *SSB bound to ssDNA (SSB-ssDNA), and the position of unbound DNA (ssDNA and dsDNA), are indicated alongside the gel. The marker DNA loaded in lane 1 of both (B) and (C) is the SmartLadder (Eurogentec).

To study the ability of *Mpn *SSB to also bind dsDNA, the protein was incubated with either single- or double-stranded φX174 DNA (Fig. 4A). While almost all ssDNA was shifted towards a lower mobility form at the lowest concentration of *Mpn *SSB tested (1.3 μM; lane 10), significant binding to dsDNA could not be observed at any protein concentration used (lanes 14–16). These results demonstrate that *Mpn *SSB preferentially binds ssDNA. Fig. 4A further shows that the gel mobility of *Mpn *SSB-ssDNA complexes becomes lower when *Mpn *SSB concentrations are increased (lanes 10–12). Similarly as described above for the oligonucleotide substrates, it is likely that increasing numbers of *Mpn *SSB molecules accumulate on the ssDNA substrates when higher concentrations of protein are present. Consequently, the size of the DNA-protein complexes will increase when the protein concentration becomes higher, resulting in lowering of the gel mobility of these complexes. Similar DNA-binding results were obtained for each of the purified, recombinant *Mpn *SSB proteins, including GST-SSB (Fig. 4B, lanes 7–11), as well as for *E. coli *SSB (Fig. 4A, lanes 2–4). Interestingly, the *E. coli *SSB protein may assemble on DNA substrates in a different fashion than does *Mpn *SSB, as more heterogeneously sized protein-DNA complexes were formed by the *E. coli *protein than by the *M. pneumoniae *protein, in particular at relatively low protein concentrations (compare lanes 2 and 10 in Fig. 4A).

To determine whether *Mpn *SSB is dependent upon divalent cations for its activity, a DNA-binding experiment was conducted in either the presence or absence of Mg^2+^. Fig. 4C shows that similar DNA-protein complexes are generated irrespective of the presence of Mg^2+ ^in the binding reaction (compare lanes 3–5 to lanes 7–9), which indicates that the protein does not require divalent cations for DNA-binding. Similarly, the SSB proteins from *E. coli *and *S. pneumoniae *were previously also shown to bind DNA irrespective of the presence of Mg^2+ ^in the binding reaction [22,34]. However, these proteins do display different binding modes in either the presence or absence of divalent cations [22,34].

### Mpn SSB stimulates E. coli RecA-promoted three-strand DNA exchange

**Figure 5:**
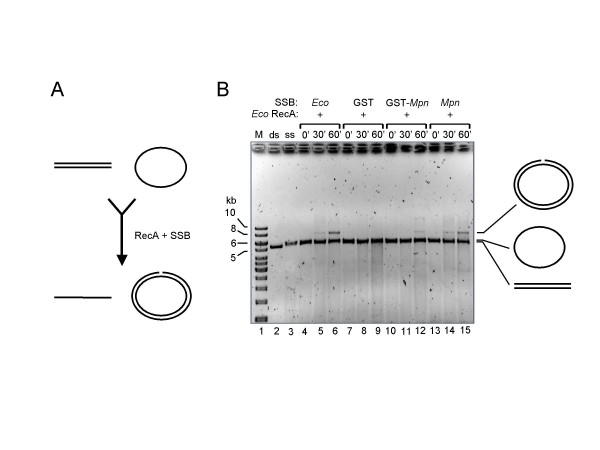
***Mpn *SSB promotes *E. coli *RecA-catalyzed three-strand DNA transfer**. (A) Schematic representation of the three-strand transfer reaction. *E. coli *RecA catalyzes the transfer of a single strand from a linear, double-stranded DNA donor molecule (at the top left) to a complementary, single-stranded, circular acceptor molecule (at the top right), resulting in a linear, single-stranded product (bottom, left) and a (nicked) circular, double-stranded product (bottom, right). This reaction is strongly promoted by the *E. coli *SSB protein. (B) *E. coli *RecA-promoted DNA strand transfer reactions using φX174 DNA. Reactions were performed at 37°C in the presence of either *E. coli *SSB (*Eco *SSB, lanes 4–6), GST (lanes 7–9), GST-SSB (GST-*Mpn*, lanes 10–12) or *Mpn *SSB (lanes 13–15). DNA concentrations used were 1 nM and 2 nM for the ssDNA and dsDNA, respectively. Reactions were terminated at either 0, 30 or 60 min of incubation (0', 30' and 60', respectively, above the lanes). The samples were separated on 0.6% agarose gels in 0.5 × TBE buffer. A black/white inverted image of an ethidium bromide-stained gel is shown. A schematic representation of the major DNA products is indicated at the right-hand side of the gel. The DNA marker (M) is the SmartLadder (Eurogentec). ds (lane 2), linear, dsDNA donor; ss (lane 3), circular, ssDNA acceptor.

Single-stranded DNA-binding proteins from several micro-organisms have previously been shown to stimulate in vitro DNA strand exchange reactions catalyzed by (homologs of) RecA. Also, the SSB proteins from *S. pneumoniae *and *D. radiodurans *were found to stimulate the activity of RecA from *E. coli *[16,21]. To study the potential of *Mpn *SSB to stimulate *E. coli *RecA-catalyzed DNA recombination, so-called three-strand exchange reactions were performed. In these reactions, RecA promotes the transfer of one of the strands of a linear, dsDNA molecule (donor) to a complementary, circular ssDNA molecule (acceptor), resulting in a double-stranded, circular product and a linear, single-stranded product (Fig. 5A).

Either alone (data not shown) or in the presence of GST (Fig. 5B, lanes 7–9), *E. coli *RecA did not detectably catalyze φX174 DNA strand exchange. However, in the presence of *E. coli *SSB, strand transfer was readily detectable after 60 min of incubation at 37°C (lane 6), while a faint DNA species at the position of the double-stranded, open circular recombination product was already visible after 30 min (lane 5). Similar results were obtained when *E. coli *SSB was replaced by either *Mpn *SSB (lanes 13–15), *Mpn *H_10_-SSB (data not shown) or GST-SSB (lanes 10–12), albeit that the latter protein was somewhat less efficient than the other recombinant proteins in stimulating RecA activity.

Taken together, these data show that *Mpn *SSB, despite having a relatively low level of sequence similarity with *E. coli *SSB, is capable of stimulating DNA strand transfer activity of *E. coli *RecA. Consequently, *Mpn *SSB may play a similar, crucial role as *E. coli *SSB in DNA recombination processes. The elucidation of the activities of *Mpn *SSB can therefore be considered an important step in unraveling the recombinatorial events that may occur in the genome of *M. pneumoniae*, and which may play a pivotal role in antigenic variation of this bacterium.

## Discussion

In order to assess whether recombination between RepMP elements within the genome of *M. pneumoniae *lies at the heart of antigenic variation of this bacterium, we set out to identify the proteins that may be involved in DNA recombination. Previously, four *M. pneumoniae *ORFs were proposed to code for proteins that could play a role in DNA recombination: MPN229, MPN490, MPN535 and MPN536 [3]. These ORFs show sequence similarity with the *E. coli *genes encoding SSB, RecA, RuvA and RuvB, respectively. Up until now, only one of these four ORFs, i.e. MPN535, has been subjected to a detailed study. This ORF was found to encode a RuvA homolog (*Mp*RuvA) which is capable of binding Holliday junctions and other branched DNA structures in a manner similar to *E. coli *RuvA [12]. In contrast to *E. coli *RuvA, however, *Mp*RuvA did not support branch migration mediated by *E. coli *RuvB. *Mp*RuvA was also found to differ from *E. coli *RuvA in its inability to allow *E. coli *RuvC to stably bind a *Mp*RuvA-bound DNA junction complex [12]. In addition, *Mp*RuvA was unable to promote DNA repair in *E. coli ruvA *mutants [12]. These findings clearly illustrated that the DNA recombination machinery in *M. pneumoniae *may differ considerably from that in *E. coli*. In agreement with this notion, we found the *Mpn *SSB protein to differ in several characteristics from its *E. coli *counterpart. First, the sequence similarity between these proteins is rather low (17% identity). Second, *Mpn *SSB and *E. coli *SSB appear to diverge in the way by which they assemble on long, single-stranded DNA substrates. This was illustrated by the heterogeneity of the DNA-protein complexes formed by *E. coli *SSB, whereas complexes formed by *Mpn *SSB appeared rather homogeneous in size (Fig. 4A). The heterogeneous size of these *E. coli *SSB-DNA complexes may be indicative of the non-random distribution of the protein on DNA substrates, reflecting the cooperative DNA-binding activity of *E. coli *SSB (see Lohman and Ferrari for a review)[22]. Nevertheless, *E. coli *SSB can interact with ssDNA in different binding modes, each having different characteristics, including cooperativity [22]. These binding modes were found to be dependent on the solution conditions, such as protein to DNA ratio, salt concentration and nucleotide sequence of the DNA substrates. Although *Mpn *SSB appears to bind with a significantly lower level of cooperativity than its *E. coli *counterpart in Fig. 4A, it is possible that cooperative binding by *Mpn *SSB is also strongly dependent on the binding conditions used. In our study, these conditions may not have been optimal for *Mpn *SSB cooperativity.

Another factor that plays an eminent role in protein-DNA complex formation is the multimeric state of the protein. *E. coli *SSB was reported to form homotetramers in solution [35,36]. The *Mpn *SSB protein also appears to exist as a tetramer in solution. With regard to the oligomeric status of *Mpn *SSB, is interesting to note that the bacterial SSBs that have previously been analyzed can be divided in separate groups on the basis of their oligomeric status and number of OB DNA-binding domains per monomer. The most prominent group consists of proteins that contain a single OB fold per monomer and function as homotetramers [18,19]. Among the members of this group are the SSB proteins from *E. coli *[32], *Streptococcus pneumoniae *[16,34] and *Herbaspirillum seropedicae *[37] and *M. pneumoniae *(this study). Another group of bacterial SSB proteins appears to possess two OB folds and function as dimers. Members of this group are the SSBs from *T. aquaticus *[20], *T. thermophilus *[20] and *D. radiodurans *[21]. These proteins are considerably larger in size than those from the first group. A common feature of both groups of SSBs is that they contain four OB folds per functional protein oligomer [21].

In contrast to the differences between the SSB proteins from *E. coli *and *M. pneumoniae *in primary sequence as well as DNA-binding characteristics, these proteins were found to be indistinguishable in their capacity to promote *E. coli *RecA-catalyzed DNA strand exchange reactions. In previous studies it has been shown that the SSBs from *D. radiodurans *and *S. pneumoniae *were also able to stimulate *E. coli *RecA protein-promoted DNA three-strand exchange reactions with at least the same efficiency as *E. coli *SSB [16,21]. However, these activities of the *D. radiodurans *and *S. pneumoniae *SSB proteins may not be regarded as unexpected, as they show, unlike *Mpn *SSB, a relatively high level of sequence similarity with their *E. coli *counterpart (38% and 31%, respectively) [16,21].

## Conclusion

We conclude that *Mpn *SSB represents the *M. pneumoniae *counterpart of the *E. coli *SSB protein. As a consequence, it is likely that this protein may be involved in various aspects of the DNA metabolism of *M. pneumoniae*. In a study on the complete proteome of *M. pneumoniae *by two-dimensional gel electrophoresis followed by mass spectrometry, the *Mpn *SSB protein was the only protein detected of the four proteins that were proposed to be involved in DNA recombination [38]. Studies aimed at the detection and characterization of the other three proteins, i.e. the RecA, RuvA and RuvB homologs, are currently underway. It will be important to determine whether these proteins interact and/or cooperate to catalyze homologous DNA recombination in *M. pneumoniae *and whether these proteins are necessary and sufficient for the recombination between RepMP elements in the bacterial genome. However, as homologs of SSB, RecA, RuvA and RuvB are found in all living organisms from bacteria to humans, it is tempting to speculate that the site-specific recombination of RepMP elements may require the activity of one or more specialized, yet unidentified proteins. The identification of such proteins and the elucidation of the DNA recombinatorial pathways in *M. pneumoniae *will be the main challenge of our future studies.

## Authors' contributions

MS cloned and sequenced the *Mpn *SSB gene, generated most of the recombinant plasmid constructs, purified the majority of the recombinant proteins, and performed most of the DNA-binding and strand transfer experiments. TH cultured *M. pneumoniae *and verified the identity of the purified recombinant proteins. CV generated some of the plasmid constructs, performed several protein purifications and did part of the DNA-binding experiments. NGH and CV designed the study and drafted the manuscript. All authors have read and approved the final manuscript.
